# Multiple defense is an effective antipredator strategy in dinoflagellates

**DOI:** 10.1093/ismeco/ycaf029

**Published:** 2025-02-14

**Authors:** Gihong Park, Hans G Dam

**Affiliations:** Department of Marine Sciences, University of Connecticut, 1080 Shennecossett Road, Groton, Connecticut 06340, United States; Department of Marine Sciences, University of Connecticut, 1080 Shennecossett Road, Groton, Connecticut 06340, United States

**Keywords:** multiple prey defense, inducible prey defense, prey defense cost and benefit, antipredator strategy, compensatory growth toxic dinoflagellate

## Abstract

Phytoplankton have evolved myriad defenses against predators; yet, studies that simultaneously test for defense fitness costs and benefits are rare. We tested for relative fitness costs and benefits of defense in the marine dinoflagellate *Alexandrium catenella* using a framework that relates growth rates of prey genotypes (strains) that differed in constitutive toxin production (low, moderate, and high) to predator (copepod) concentration. Our approach is based on a novel molecular technique that allows one to disentangle the effect of predation mortality from the cell growth reduction due to toxin production. Results show that the strain with the highest constitutive toxin production was the only one that expressed inducible toxin production—a strategy that paid off as its fitness benefit outweighed its cost. Surprisingly, the moderate toxin strain that derived the highest relative fitness benefit increased cell division rate (akin to compensatory growth) and decreased cell size, while keeping its volume-specific toxin production constant in response to predation. These results suggest an effective antipredator defense portfolio.

## Introduction

Phytoplankton, which are the basis of pelagic food webs and represent 50% of the global primary production [1, 2], have evolved myriad antipredator defense traits, including escape, armored cell walls, spines, altering cell size, colony formation, and toxin production [3–9]. For these defenses to persist their benefit must outweigh their costs. Yet, with few exceptions, documenting the cost of inducible defense in phytoplankton has proved elusive [10, 11], and documenting the simultaneous fitness benefit and cost of phytoplankton prey defense is rare [11–13].

Toxic algal blooms have become a global threat, threatening marine food webs, public health, and coastal fisheries economies [14, 15]. Bioaccumulation of paralytic shellfish toxins (PST), the best studied group of toxins in species of the dinoflagellate genus *Alexandrium* [16, 17], is documented throughout the food web [18–20]. *Alexandrium* species can produce PST, as well as spirolides, gymnodimines, goniodomins, bioactive extracellular compounds (BECs) including reactive oxygen species (ROS) [21–23]. Altogether, these compounds have demonstrated antagonistic effects on protistan competitors or consumers [23–27]. Ingestion of toxigenic *Alexandrium* spp. reduces ingestion rate [28–30], decreases development and offspring production [31–33], and reduces population fitness [34] in metazoans. Some *Alexandrium* species, in turn, respond to predators by increasing toxin production [11, 30, 35], bioluminescence [36], or by splitting into single cells or shorter chains, which reduces swimming velocity and potentially encounter rates with predators [37, 38]. Induced toxin production also deters predation [12, 13, 28, 35]. Altogether, these responses are interpreted as defense mechanisms.

The Optimal Defense Theory, mostly tested on higher plants, is based on the fundamental assumption that defenses are beneficial, yet costly [39]. Such costs are also hypothesized to explain why prey express intermediate instead of maximum levels of defense [40]. In freshwater phytoplankton communities there is evidence for selection of high growth species with intermediate levels of defense, but the degree of defense increases with increasing predator concentration [41].

Recently, a direct fitness cost of predator-induced toxin production (reduction in cell division rate) in toxigenic dinoflagellate *Alexandrium catenella* has been shown by Park and Dam [11] using a novel approach that measures relative gene expression (RGE) of a cell growth marker (that correlates to cell division rate) in the presence and absence of predator*.* This approach is useful in disentangling the reduction in cell growth rate due to predator-induced toxin production from the cell losses due to predation and obviates the need for experiments that use indirect predator cues to study the cost of defense [12, 13, 41].

**Figure 1:**
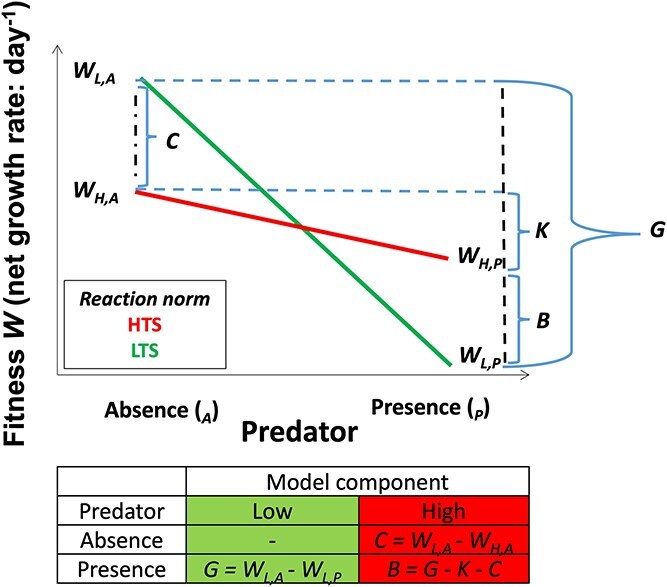
A graphical model of prey fitness differing in the expression of defense as a function of predation. The model was modified from Simms and Rauscher [35] and Simms [36]. For simplicity, the figure only shows two genotypes. Calculations of the model term for two different genotypes; the *L* and *H* refer to low toxin strains (LTS) and HTS in the absence (*A*) and presence (*P*) of predator in terms of fitness. Fitness (*W*) represents the net growth rate. *C* = the cost associated with defense for the defended genotype relative to the susceptible genotype, *G* = the reduction of fitness due to predation in the susceptible genotype, *K* = the reduction of fitness due to predation in the defended genotype, and *B* = the benefit of defense.

Here, we measured the simultaneous fitness costs and benefits in response to predator concentration among *A. catenella* strains that vary in their constitutive and inducible PST production (Fig. 1). We slightly modified extant models [42, 43] to compare fitness among prey that varied in their degree (low, moderate, and high) of toxin production. In the model calculations, we disentangled the direct fitness cost of predator-induced toxin production from predation mortality using RGE of cell growth [11]. In the process, we also observed a case akin to compensatory growth [44–46], which suggests that the dinoflagellate effectively employed multiple antipredator mechanisms.

## Materials and methods

### Sample collection and culture

The three strains used in this study were previously classified as *Alexandrium tamarense* complex Group I, and since then reclassified as *A. catenella* [47]. A low toxin strain GTCN-16 (LTS: ~5 femtomole [fmol] cell^−1^, isolated from Mumford Cove, Groton, CT, USA), a moderate toxin strain CB-307 (MTS: ~20 fmol cell^−1^, Casco Bay, ME, USA), and a high toxin strain BF-5 (HTS: ~30 fmol cell^−1^, Bay of Fundy, Canada) were grown in F/2 medium without silicate [48]. These cultures have kept their constitutive toxin content rather constant for at least two decades as evidenced by comparison to our own previous studies [27, 49]. All experiments were conducted in an environmental chamber kept at 18°C, illuminated with fluorescent lighting (~100 μmol m^−2^ s^−1^) set to a 12 hr:12 hr light:dark photoperiod.

The calanoid copepod *Acartia hudsonica,* which served as the predator, was collected from Eastern Point Bay, CT, USA (41°18′47.6″N 72°03′50.2″W). Cultures were maintained with a mixed diet of three phytoplankton species, *Tetraselmis* sp., *Thalassiosira weissflogii*, and *Rhodomonas* sp., for at least three generations to remove maternal and environmental effects [50] under the same temperature and light conditions as the prey. Before assays, copepods were acclimatized to experimental conditions for 24 h and starved during that period to ensure complete gut evacuation [51].

### Susceptibility to predator concentration

The three strains of *A. catenella* were placed in 500 ml bottles (200 cells ml^−1^) either in the absence (controls: constitutive toxin production) or with varying predator concentration (inducible toxin production: 10, 20, 40, and 80 ind. L^−1^) of adult female *A. hudsonica* for two days. The predator-cell ratios in the experimental enclosures are orders of magnitude lower than what is observed under field conditions; thereby, minimizing biases arising from predator nutrient recycling (excretion) or defecation on cell growth or toxin production [30, 52]. Experiments were done in five replicate controls and treatments and carried out at 18°C in a walk-in environmental chamber under the nutrient replete condition (F/2 medium − Si). At the end of the assays, copepods including nauplii, eggs, and fecal pellets were gently removed by wet-sieving onto a 63 μm mesh. Two separate aliquots (245 ml) from each bottle of *Alexandrium* cells were filtered onto 5 μm pore size polycarbonate membranes, one for toxin analysis and another for ribonucleic acid (RNA) extraction. For the former, the filter was carefully washed off with 1 ml of 0.1 M acetic acid, and for the latter 1 ml of TRIzol Reagent (Invitrogen), then stored at −80°C until analysis. An aliquot of 10 ml was also preserved in 0.5% acid Lugol’s solution for cell counting and size measurement. Cell sizes were measured using NIS-Elements AR 3.0 software, Nikon.

### Toxin analysis and growth rate

Samples for toxin analysis were treated as described by Park and Dam [11]. Briefly, homogenized cells in 0.1 M acetic acid (MP Biomedicals) were filtered through 0.45 mm ultra-filtration centrifuge cartridges (Millipore) to remove broken thecal plates. PST content was determined by reverse-phase ion-pairing HPLC using the post-column oxidative fluorescence method [53]. Saxitoxin congeners, gonyautoxins 1 through 4 (GTX1–4), saxitoxin (STX), neosaxitoxin (NEO), C1, and C2 were identified. Fluorescent PST derivatives were quantified by comparison to certified toxin standards from the National Research Council of Canada (Halifax). The final toxin content was calculated from the chromatograms and integrated peak areas (Empower, Waters), and expressed as fmol unit (fmol cell^−1^). Cells of *A. catenella* preserved in Lugol’s solution were counted on an inverted microscope (Olympus IX70 Model). The net cell growth rate (*μ*) was calculated, assuming exponential growth.

### Ribonucleic acid extraction and complementary deoxyribonucleic acid synthesis

Cells from the samples preserved in TRIzol were thawed in ice and homogenized as described above. The tubes were then centrifuged at 10 000 × g for 1 min and the supernatant was transferred to a clean tube for subsequent RNA extraction. Total RNA was isolated as described previously [54] and quantified using a NanoDrop ND-1000 spectrophotometer (Thermo Fisher Scientific). Complementary deoxyribonucleic acid (cDNA) was synthesized from 180 to 280 ng of the total RNA using iScript™ cDNA Synthesis Kit (BioRad) and modified oligo dT [55] and potential genomic DNA contaminant was removed by DNase in the process. The resultant first-strand cDNA was purified using Zymo DNA Clean and Concentrator (Zymo Research).

### Gene expression analysis

Gene expression analysis was conducted using reverse transcription quantitative PCR (RT-qPCR), as in Park and Dam 2021 [11], targeting saxitoxin biosynthesis gene (*sxtA4*) and a mitotic cyclin B gene (*cyc*) in *A. catenella,* a biomarker for cell growth rate. Quantitative PCRs (qPCRs) were performed on a StepOnePlus™ real-time PCR system and run in 10 μl reactions with Fast SYBR® Green Master Mix (Applied Biosystems) with the following amplification conditions: a 10 min activation step at 95°C, followed by 45 cycles of denaturation for 15 s at 95°C, annealing for 30 s at 60°C, and extension for 15 s at 72°C. Then, a melting-curve analysis was performed to check for nonspecific product formation. Due to more stable gene expression in *lbp* (luciferin-binding protein) than *cob* (cytochrome b), the former was used to normalize the gene expression level [11]. Expressed levels of the target genes were compared to the reference gene according to the Pfaffl method [56] corrected by standard curves to estimate the primer efficiency (see the electronic Supplementary Information S1).

### Relative fitness cost and benefit of defense

To quantify the relative costs and benefits of antipredator defense, we used the model of Simms and Rausher [42] and modified the concept behind the equations in graphical representation (Fig. 1). The illustration considers a defended genotype (high toxin strain, HTS: *_H_*) and susceptible genotype (low toxin strain, LTS: *_L_*) using reaction norms to compare genotype fitness as a function of predator absence/presence, which can be extended to a predator concentration (none to high). In the absence of predation, the model purposefully assumes that toxin production is constitutive and the difference between the fitness of LTS and HTS represents the relative fitness cost (*C*) of constitutive defense (toxin production) for the defended genotype:


(1) \begin{equation*} C={W}_{L,A}- {W}_{H,A}. \end{equation*}

*W_L,A_* and *W_H,A_* are growth rates (representing fitness) of LTS and HTS in the predator-absent environment, respectively. The reduction in fitness of the LTS due to predation (*G*) is given by the difference in fitness when predators are absent (*W_A_*) and present (*W_P_*), respectively:


(2) \begin{equation*} G={W}_{L,A}- {W}_{L,P}. \end{equation*}

By similar reasoning, *K* is the reduction in fitness in the HTS due to predation:


(3) \begin{equation*} K={W}_{H,A}- {W}_{H,P}. \end{equation*}

Theoretically, *K* will range from zero to *G*; zero occurs when the defense is perfectly effective, and *G* is the upper limit of *K*, the point at which the defense is fully ineffective.

The relative fitness benefit of the predator-induced defense (*B*) to the HTS is:


(4) \begin{equation*} B=G- C- K \end{equation*}

where 0 < *B* < *G*. Due to the direct fitness cost of predator-induced toxin production within *K* [11], Equation 4 can be substituted into the effect of cost, and yields:


(5) \begin{equation*} B=G- C- \left(K- fitness\kern0.17em cost\kern0.17em of\kern0.17em toxin\kern0.17em production\right). \end{equation*}

### Effect of prey on predator ingestion

These ancillary experiments were designed to test if toxic prey reduced predator ingestion rate. We used fecal pellet production, which is strongly correlated to copepod ingestion rate [57], as a proxy for ingestion rate. For each treatment, 40 pairs of newly matured females and males were placed into 20 ml petri dishes for 2 days (*n* = 160), which were fed at a concentration of 800 μgC L^−1^ of control (mixed-diet of *Tetraselmis* sp., *T. weissflogii*, and *Rhodomonas* sp.) and the three *Alexandrium* diets. The toxic diet treatments corresponded to a predator concentration of 20 ind. L^−1^, yielding a predator:prey ratio of 0.0002, again orders of magnitude lower than under field conditions. Each treatment was divided into two groups and peroxidase (1.25 μg ml^−1^) was added to the second group to test ROS effect on copepods because ROS produced by the *A. catenella* strains of this study are linked to high mortality rate of ciliates and heterotrophic dinoflagellates [27]. Pellet production rate was estimated as in Besiktepe and Dam [57]. All experiments were done under the same conditions of light, temperature, and light:dark photoperiod for the phytoplankton cultures.

### Statistical analysis

After checking for normality and heteroscedasticity, a two-way analysis of variance (ANOVA) was performed to test the interaction of strain and predator concentration on cell toxin content, cell growth rate, and gene expression. A one-way ANOVA was used to test susceptibility of the three strains vs predator concentration for growth rate, cell size, PST content, and relative expression of genes related to PST production and growth (five independent replicates for growth rate and three samples for PST analysis and gene expression). Post-hoc Tukey HSD of means compared parameters within groups. A comparison of slopes of net growth rate vs predator concentration among the strains was done with ANCOVA. Predator-dependent changes in gene expression were also tested separately by regression analysis and the Akaike Information Criterion (AICc) was used for the best-fit regression model selection. One-way ANOVA tested for effects of the strains on predator fecal pellet production rate. A *T*-test was used for the effect of the enzyme peroxidase on traits (predator egg production and ingestion) relative to each treatment without peroxidase. All analyses were performed using SigmaPlot version 11.0 and SPSS version 29 software.

## Results

### Toxin content

**Figure 2:**
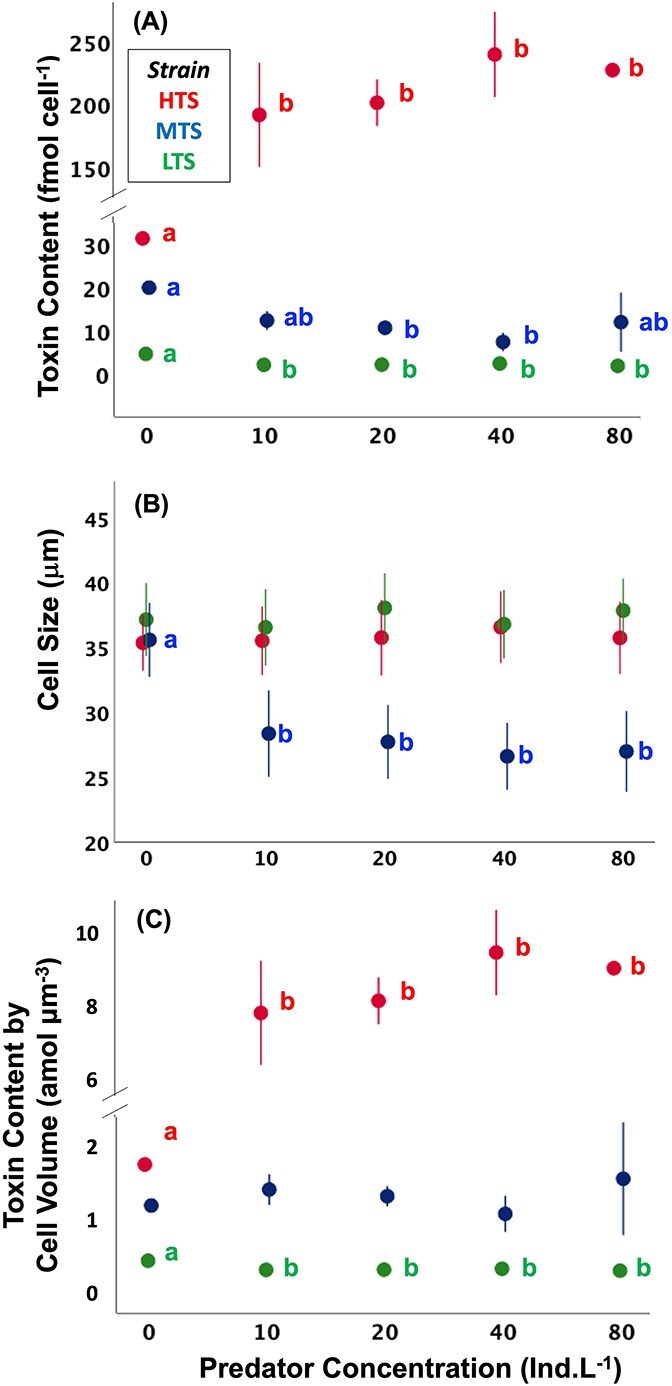
Cellular toxin content (A), cell diameter (B), and volume-specific cellular toxin content (C) of *Alexandrium catenella* strains as a function of predator concentration. Colors indicate strains and letters above bars represent significant statistical differences between mean values of groups compared to control (0 ind. L^−1^) and among treatments. Error bars represent ±1 standard deviation of the mean (*n* = 3). The *Y*-axis was truncated for clarity.

The pattern of cellular toxin content vs predator concentration differed with prey strain (Fig. 2; Supplementary Information S1; two-way ANOVA, *F_4,30_* = 681, *P* < .001). In the LTS and the MTS strains toxin content was higher in the absence of predators and was relatively constant in the presence of predators. In the former, toxin content decreased from (4.6 in the absence of predator to ~2.1 fmol cell^−1^ in the presence of predators (Fig. 2A; ANOVA, post-hoc Tukey HSD, *F_4,10_* = 83.2, *P* < .001), while in the latter the decrease was from 20 ~ 12 fmol cell^−1^ a (Fig. 2A; ANOVA, post-hoc Tukey HSD, *F_4,10_* = 5.53, *P* = .013). By contrast, toxin content in the HTS increased with predator concentration by up to 684% from 31 to 215 fmol cell^−1^ (Fig. 2A; ANOVA, Post-hoc Tukey HSD, *F_4,10_* = 33.7, *P* < .001). Predators caused a reduction of cell size in the MTS (Fig. 2B; ANOVA, Post-hoc Tukey HSD, *F_4,567_* = 1820, *P* < .001), but not in the other two strains. However, the volume-specific toxin content (amol μm^−3^) was not affected by predators (Fig. 2C: ANOVA, Post-hoc Tukey HSD, *F_4,10_* = 0.731, *P* = .591).

### Prey net growth rate

**Figure 3:**
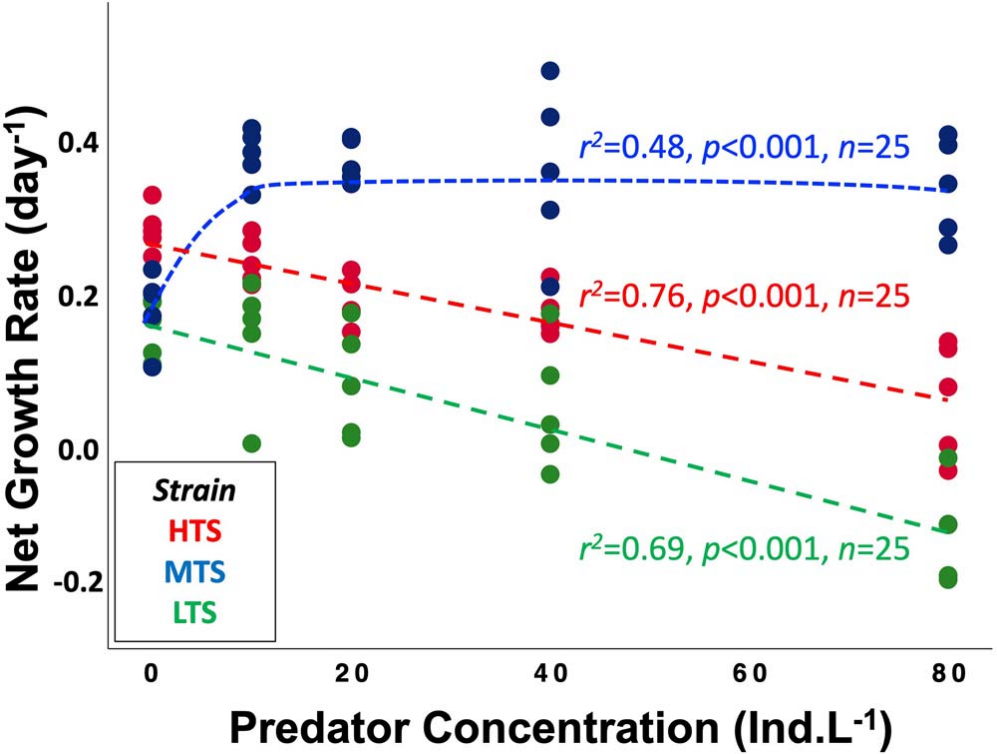
Net growth rate versus predator concentration of three strains of *Alexandrium catenella*. Colors indicate strains and the lines represent fits from the shown regressions.

The prey net growth rate was a function of both strain and predator presence (Supplementary Information S2; two-way ANOVA, *F_2,60_* = 71.5, *P* < .001, and *F_4,60_* = 8.8, *P* < .001). There was an interaction of strain and predator concentration on net growth rate (*F_8,60_* = 6.2, *P* < .001). The MTS increased growth with predator concentration up to 10 copepods L^−1^, and then leveled off (Fig. 3; MTS: y = 0.17+$\frac{\ 0.21x\ }{5+x}$). By contrast, net growth rates in the other two strains decreased with predator concentration, but a higher slope for the HTS (Fig. 3; ANCOVA for the difference of slope, *P* < .001; HTS: y = 0.26–0.003x and LTS: y = 0.15–0.004x). Hence, the LTS was the most susceptible strain to predation.

### Gene expression of toxin production and cell growth markers

**Figure 4:**
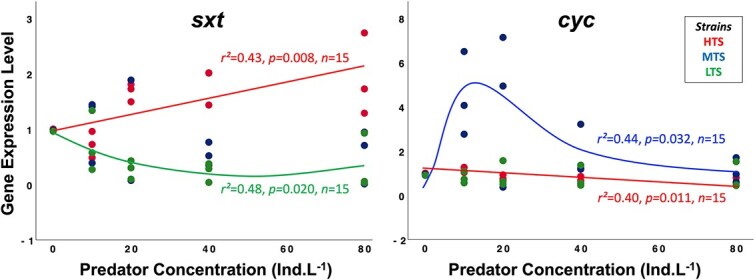
Mean (±1 SD, *n* = 3) RGE level of STX gene (A; *sxtA4*) and cell growth-related gene (B; *cyc*) versus predator concentration in strains of *Alexandrium catenella.* Colors indicate strains and lines are regression fits.

The RGE of *sxtA4* vs predator concentration was strain-dependent (Fig. 4A). Gene expression was strongly upregulated with increasing predator concentration in the HTS (y = 1.03 + 0.013x; ANOVA, *F_1,13_* = 9.69, *P =* .008), independent of predator concentration in the MTS (ANOVA, *F_1,13_* = 1.67, *P =* .219), and decreased slightly then then leveled off in the LTS (y = 0.98–0.034x + 0.001x^2^; ANOVA, *F_1,13_* = 5.54, *P =* .020). RGE of *cyc* also depended on strain (Fig. 4B). It was independent of predator concentration in the LTS (ANOVA, *F_1,13_* = 0.18, *P =* .678), but decreased in the HTS (y = 0.02–0.005x; ANOVA, *F_1,13_* = 8.65, *P =* .011). Surprisingly, RGE of *cyc* peaked at 10–20 ind. L^−1^ and then decreased (y=$89{e}^{-0.5{\left(\frac{\mathit{\ln}\left(\frac{x}{33}\right)\ }{1.02}\right)}^2}$; ANOVA, *F_2,12_* = 4.66, *P =* .032).

### Relative fitness cost and benefit of defense

**Figure 5:**
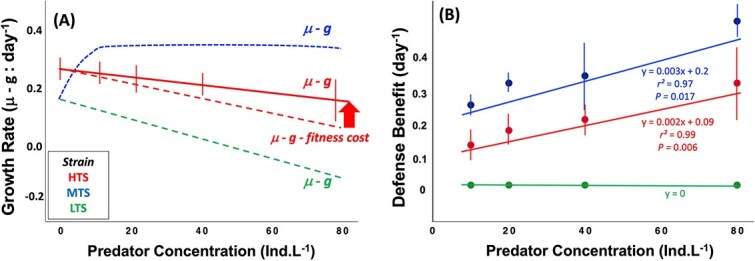
Growth rate (A) and relative defense benefit (B) versus predator concentration. The net growth rate is recalculated based on the effect of the direct fitness cost due to inducible toxin production in the high toxin strain. In panel B, the defense benefit is evident in the moderate and high toxin strains and increases with predator concentration. Colors indicate strains. Error bars represent ±1 standard deviation of the mean (*n* = 5).

The relative costs and benefits of defenses are summarized in Fig. 5 and Table 1. A significant cost to constitutive toxin production would have been evident if growth in the LTS was higher in the absence of predators than in the other two strains (Fig. 1). Yet, growth rate of the LTS was lower than the HTS when predators were absent (Supplementary Information S3). Thus, the relative fitness cost (*C*) associated with constitutive toxin production appears to be negligible (Table 1; *C_H_*: -0.14, ANOVA, *F_2,12_* = 5.43, *P =* .021). Losses related to predation (*G*), the reduction of fitness due to predator consumption, increased with predator concentration (0.01 to 0.28 d^−1^). *K* values (inverse of fitness change with predator concentration) differed among the strains. Theoretically, *K* will range from zero (effective) to *G* (ineffective); however, negative *K* values in the MTS mean a fitness gain (benefit) with predator concentration. Thus, the MTS and HTS derived a fitness benefit (*B*) relative to the LTS. (Fig. 5B; *r^2^* = 0.97, *P* = .017, *n* = 4). The calculation of benefit for the HTS (Eq. 4) had to consider the direct fitness cost due to predator-induced toxin production (Eq. 5); thus, equation 6 became *B* = *G* + *C* – (*K – fitness cost of toxin production*) to account for the apparent negative value of the *C* term (Supplementary Information S3 and Fig. 5B; *r^2^* = 0.99, *P* = .006, *n* = 4).

**Table 1:** Relative fitness cost and benefit of defense in the three strains of *Alexandrium catenella* (LTS, MTS, and HTS) with predator concentration. Abbreviations represent the relative fitness cost of constitutive toxin production (*C*), the reduction of fitness due to predation (*G*), and the benefit of defense (*B*), expressed per day unit (day^−1^).

	Concentration	Model component					
Predator	(ind. L^−1^)	LTS		MTS		HTS	
Absence	0	*C_L_*	0	*C_M_*	0	*C_H_*	-0.14
Presence	10	*G_10_*	0.01	*B_M10_*	0.22	*B_H10_*	0.11
Presence	20	*G_20_*	0.08	*B_M20_*	0.28	*B_H20_*	0.15
Presence	40	*G_40_*	0.11	*B_M40_*	0.30	*B_H40_*	0.18
Presence	80	*G_80_*	0.28	*B_M80_*	0.45	*B_H80_*	0.28

### Effect of prey on predator ingestion

**Figure 6:**
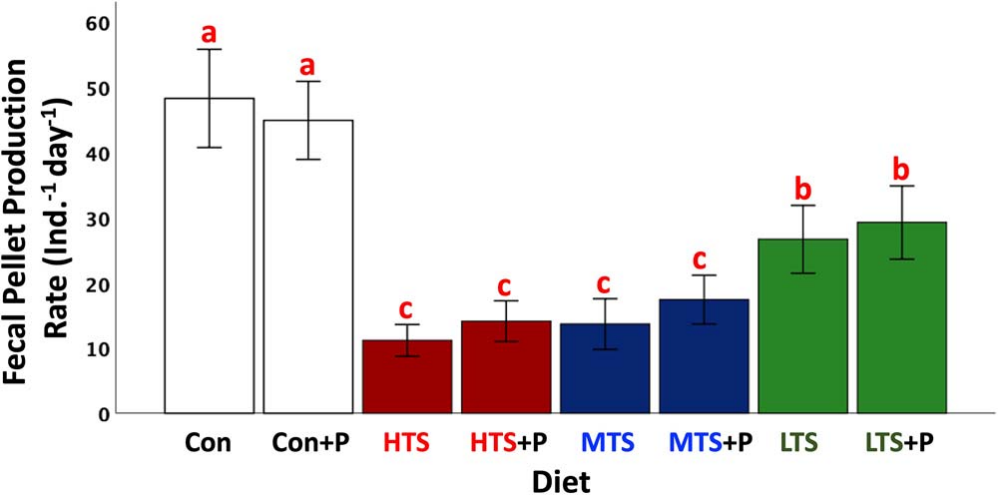
Fecal pellet production rate of copepod predator female *Acartia hudsonica* fed control (nontoxic diet) and toxigenic strains of *Alexandrium catenella.* +P represents the addition of the enzyme peroxidase to scavenge ROS from the prey. Colors indicate prey strains and letters above bars show significant differences between mean values of groups compared to the control diet and among treatments. Error bars represent 95% confidence interval (*n* = 20).

The fecal pellet production rate on all toxigenic diets was lower than on the nontoxic diet, with the MTS (71%) and HTS (77%) showing the greater declines, suggesting lower predator ingestion on these diets than nontoxic diet (Fig. 6; ANOVA, Post-hoc Tukey HSD, *P* < .001). Furthermore, the MTS and HTS also showed lower fecal pellet production than the LTS. By contrast, the addition of the enzyme peroxidase (+P) had no effect on fecal pellet production relative to treatments without peroxidase (Fig. 6; *T*-test, *P* > .05), suggesting no obvious deleterious effect of *Alexandrium* ROS on the predator.

## Discussion

Multiple defenses have been documented in dinoflagellates, but the simultaneous costs and benefits of these defenses have received scant attention. We estimated the relative fitness costs and benefits of antipredator defense using a modification of previous conceptual models in a toxic dinoflagellate. We confirmed that inducible toxin production pays a fitness benefit. In the process, we discovered a response akin to compensatory growth, which also acts as an effective antipredator mechanism. Below we discuss these mechanisms and some challenges of studying multiple defenses.

### Toxin production

The optimal defense theory posits that allocation of resources to defenses results in a trade-off between defense traits and other traits such as growth. Thus, our original intent was to compare and contrast the costs and benefits of three strains of *A. catenella* that differed in their degree of toxin production. If there was a significant relative fitness cost to constitutive toxin production, the growth rate of the HTS should have been the lowest among the three strains in the absence of predators (Fig. 3). Yet, this was not the case (Table 1) suggesting the relative constitutive cost of toxin production was small or negligible. The ANCOVA analysis also revealed that the LTS showed the greatest depression in net growth rate, demonstrating the disadvantage of lower toxin production (Fig. 3).

Our study confirms the observation of predator-induced toxin production for the HTS [11, 30, 35], which increased toxin content up to 684% in proportion to predator concentration, leading to a direct fitness cost and a reduction in cell division rate—a classic trade-off between defense and growth [11]. The LTS in this study, which had been reported earlier as a non-toxigenic strain [27, 30], showed small but measurable PST content, but predators did not induce additional toxin production. Likewise, predator-induced toxin production was not evident in the MTS. Rather, unlike the other two strains, the predator induced a sizable reduction of cell diameter and volume. Yet, volume-specific toxin production in the MTS did not respond to predator concentration. The disparate results among the three strains add complexity to predictions of expression of predator-induced toxin production and suggest that broad generalizations of the cost of chemical defense even within the same species can be perilous.

Park and Dam [11] showed that investment in predator-induced production of genes involved in the first step of saxitoxin biosynthesis also resulted in a reduction of the RGE of the cell growth-related gene (*cyc*), equivalent to a mean decrease of the cell growth rate of 32% (range: 17–39%) under a similar predator–prey ratio to this study. The same method was applied here to separate the direct fitness cost of toxin production from cell loss by predation. The levels of RGE of *sxtA4* increased significantly with predator concentration in the HTS, consistent with the independent measures of toxin content. In contrast, the RGE of *cyc* decreased significantly with predator concentration in the HTS, indicating the fitness cost due to inducible toxin production resulting in a reduction of cell division rate of 37% (range: 22 to 42%); altogether these two studies suggest a rather constant cost of inducible toxin production under nutrient and light-replete conditions. The LTS, in which toxin production was not induced by predators, showed no statistically significant reduction in the RGE of *cyc* between the absence and presence of predators. The RGE of *cyc* in the MTS increased four-fold in the presence of predators, consistent with an increase in the cell division rate, which was reflected in the increase in net growth rate (~0.4 d^−1^). However, this latter strain did not display up-regulation in RGE of *cyc* at high predator concentrations (40 and 80 ind. L^−1^). Thus, a future test should be conducted with other growth-related genes such as the chloroplast *rbcL* gene, which encodes for RuBisCo.

A recent model to estimate costs and benefits of PST production suggested that toxin inducibility is high in the presence of predator cues and dependent on substrate (light intensity and nutrient) supply [58]. Direct constitutive costs of toxin production along resource gradients also appear to be variable [59]. The present study and another one [11] in which a cost of defense was evident were done under nutrient replete conditions. Preliminary tests from our study show that resource (light) limitation (~10 μmol m^−2^ s^−1^) does not change the pattern of net growth rate vs predator concentration among the three strains (Supplementary Information S4). Furthermore, the differences in constitutive toxin content among the strains of this study are also invariant across experiments conducted over two decades [27, 49]. Altogether, these observations suggest that the differences in growth rates and toxin contents among the strains and the patterns in response to predators are robust.

### Predator responses

The fecal pellet production rates of copepod predator fed the two toxigenic diets with HTS and MTS were lower than the diets with LTS and nontoxic control prey (HTS = MTS < LTS < Control). Because fecal pellet production is strongly correlated to copepod ingestion rate [57], we infer that the more toxic prey reduced predator ingestion rate regardless of the addition of the enzyme peroxidase. Even though ROS seem to be linked to the toxicity of the *Alexandrium* spp. to ciliates and heterotrophic dinoflagellates [27], our result showed there were no significant effects of ROS on predator ingestion, suggesting that ROS are not deleterious to copepods. Cell size observations revealed that 10% of MTS cells with reduced volume consisted of 2-cell chains even under the highest predator concentration, whereas no chain formation was observed in the other two strains. The reductions in cell size and increase in growth rate of the MTS are analogous to observations on *Alexandrium minutum* (strain GUMACC 83) exposed to chemical cues of the copepod *Calanus finmarchicus* [12]. A similar reduction in cell volume in response to predator cues has been observed in diatoms, which is linked to increases in cellular biogenic silica and thickening of the wall, consisting with a defense strategy [60]. Selander *et al.* [37] demonstrated a reduction in the swimming speed of *A. tamarense* exposed to copepod cues due to large chains splitting into smaller ones or single cells. The predator-induced breakup of chains allows cells to enter a “stealth mode” and reduce encounter rates with predators, lessening mortality [37]. In addition, video sequences analysis of toxigenic *Alexandrium* spp. shows evidence of cell rejection by copepods, consistent with a benefit of chemical defense [12, 61]. While some degree of rejection of the high toxin strain of *A. catenella* by copepods occurs [30], there is also evidence of physiological incapacitation post ingestion [62]. Thus, chemical defense and reduction in cell size tend to decrease predation rate. But the increase in growth rate in the MTS strain would, over time, result in increased encounter rates. We estimated the total volume of the MTS using the mean cell number (Supplementary Information S5). The estimated total volume vs predator concentration is the reverse of the observed for net growth rate vs predator concentration. There is a change between the control and the treatments, but not among the treatments. If encounter rate is driven by cell volume, then contact rate could be less in the treatments than the control. Thus, this reduction in encounter rate could be offset by higher cell concentrations resulting from the increase in cell growth rates. Whether the reduction in fecal pellet production on the MTS and HTS resulted from lower ingestion rate, cell rejection, or lower encounter rates remains to be explored in further studies.

### Compensatory growth

The positive fitness benefit with increasing predator concentration has important implications for bloom dynamics and modeling predator–prey interactions. Traditionally, predation is considered strictly a fitness loss for phytoplankton [63–66]. However, indirect positive effects of predation on HAB development have been hypothesized [59, 67]. In the present study, the observed effect in the MTS is direct, which implies that *A. catenella* can not only compensate for predation losses but also increase its growth rate in response to predation. This increase in growth is consistent with compensatory growth, which is posited as a tolerance mechanism for the deleterious effect of herbivory on vegetative and reproductive tissue in higher plants, where predation does not necessarily lead to death [44–46]. In our study, the increased cell division may have been linked to the reduction in cell size in response to predation. Perhaps selection for duplet cells led to singlets, which grew faster (consistent with increases in RGE of *cyc*). It is also possible that reduction in cell size in response to predators reduced ingestion rates leading to higher net growth rate. In any case, this was still a benefit to the prey. Further study is required to assess the relative role increased cell division or decreased cell loss leading to decreased predation rates in determining the prey net growth rate.

The relative fitness cost/benefit analysis revealed that the most toxigenic strain did not derive the highest fitness benefit in response to predator concentration, implying that inducible toxin production is not always the best defense mechanism. Rather, the best defended strain retained moderate volume-specific toxin production independent of predator concentration. This same strain is the one that exhibited compensatory growth suggesting that compensatory growth, a tolerance mechanism, is also an effective antipredator strategy.

The results suggest that the direct fitness cost of predator-induced toxin production may provide a feedback mechanism that constrains blooms of high toxin strains (HTS; inducible defense in this study). However, predators could enhance blooms of moderate toxin strains (constitutive, but structural-chemical defense in this study) of *A. catenella* via compensatory growth. Thus, further understanding of antipredator defense and toxic dinoflagellate bloom dynamics will require examining multiple defense mechanisms that affect both cell division rate and predation losses.

### Limitations of the study and challenges for future research

Our study highlights some challenges of testing for benefits and costs of prey defense that extend beyond our own experiment. In our study, the prey defense was not always proportional to predator density. This may reflect a saturation of the defense response or constraints on the plastic expression of the defense. In addition, ideally a test would be a comparison of strains that express only one trait such as inducible toxin production. Here, it became evident that a single strain may express multiple defense. There are, in addition, other forms of defense that could have been expressed but were not measured. For example, *Alexandrium* species have been known to produce BECs and allelopathic compounds that affect competing natural or cultured algal cells and protist or metazoan predators [23, 62, 68]. These allelochemicals released by *Alexandrium* cells have various effects, i.e. lysis related to ichthyotoxic and hemolytic properties, that may also affect all kinds of predators in addition to toxins [24, 25, 27, 69]. Furthermore, bioluminescence occurs in 89% of screened *Alexandrium* species [70], which is also considered a defense mechanism [36, 71, 72]. Thus, if more than one defense trait is expressed (a defense portfolio), there may be multiple trade-offs that would need to be measured [73], and which would be environment-dependent. Although all the effects of these defense mechanisms would be manifested in the measurement of *G* (predation), one cannot be certain that they are manifested in all compared strains. Failure to consider multiple defensive traits could also account for perceived benefits of a defense trait without costs in *Alexandrium* species [12, 29, 38], cyanobacteria [74], and other taxa [75, 76].

## Supplementary Material

Supplementary_Information_(2-9-2025)_ycaf029

Data_(2-5-2025)_ycaf029

## Data Availability

The data are publicly available without restriction at the time of publication.
