# Supplementary material for: Multiple defense is an effective antipredator strategy in dinoflagellates
Source: ISME Commun. 2025 Feb 14;5(1):ycaf029. doi: 10.1093/ismeco/ycaf029 (PMC11894931; doi:10.1093/ismeco/ycaf029)
Supplement: Supplementary_Information_(2-9-2025)_ycaf029 [file supplementary_information_(2-9-2025)_ycaf029.pdf]

Supplementary Information

S1.

qPCR efficiency (10 $^{(-1/slope)}$), R2 value (r^2), and melting temperature (Tm: °C) of the investigated *Alexandrium catenella* strains.

Strain	Function	Gene	qPCR efficiency	R2 value	Tm
BF-5	Toxin	*sxtA4*	1.899	0.997	85.8±0.30
	Growth	*cyc*	1.752	0.994	85.3±0.31
	Reference	*lbp*	2.008	0.995	82.7±0.24
CB-307	Toxin	*sxtA4*	1.817	0.998	85.8±0.35
	Growth	*cyc*	1.659	0.996	85.3±0.25
	Reference	*lbp*	1.978	0.998	75.6±0.21
GTCN-16	Toxin	*sxtA4*	1.812	0.997	71.3±0.23
	Growth	*cyc*	1.750	0.997	85.5±0.48
	Reference	*lbp*	2.350	0.921	82.2±0.21

S2.

Two-way ANOVA results for cell toxin content, net growth rate, and relative expression of STX-related gene (*sxtA4*) and cell growth-related gene (*cyc*) during the experiment. Factors in the ANOVA are of three *Alexandrium catenella* strains, predator concentration (five levels: 0, 10, 20, 40, and 80 ind. L^{-1}), and the interaction of strain and predator concentration. SS(III), type III sum of squares; df, degrees of freedom; MS, mean sum of squares for ANOVA; *F*, statistic for ANOVA test; *p*, the significance of the ANOVA test; η^2, partial eta squared.

Cell Toxin Content (fmol $cell^{-1}$)						
Source	SS(III)	df	Mean Square	*F*	*p*	η^2
Strain	292158	2	146079	681	0.001	0.98
Predator Concentration	24859	4	6215	29	0.001	0.79
Strain × Predation	60692	8	7587	35	0.001	0.90
Residual	6437	30	215			
Total	571106	45				
**Net Growth Rate (d^{-1})**						
Source	SS(III)	df	Mean Square	*F*	*p*	η^2
Strain	1.04	2	0.517	71.5	0.001	0.70
Predator Concentration	0.256	4	0.064	8.84	0.001	0.37
Strain × Predation	0.360	8	0.045	6.22	0.001	0.45
Residual	0.434	60	0.007			
Total	3.99	75				
**Relative gene expression (*sxtA4*)**						
Source	SS(III)	df	Mean Square	*F*	*p*	η^2
Strain	0.949	2	0.475	120	0.001	0.8
Predator Concentration	0.231	4	0.058	14.8	0.001	0.49
Strain × Predation	0.331	8	0.041	10.5	0.001	0.58

Source	SS(III)	df	Mean Square	F	p	η^2
Residual	0.237	60	0.004			
Total	4.217	75				

Relative gene expression (*cyc*)

Source	SS(III)	df	Mean Square	F	p	η^2
Strain	26.8	2	13.4	11.0	0.001	0.42
Predator Concentration	12.1	4	3.03	2.49	0.064	0.25
Strain × Predation	21.8	8	2.72	2.24	0.053	0.37
Residual	36.5	30	1.22			
Total	187	45				

S3.

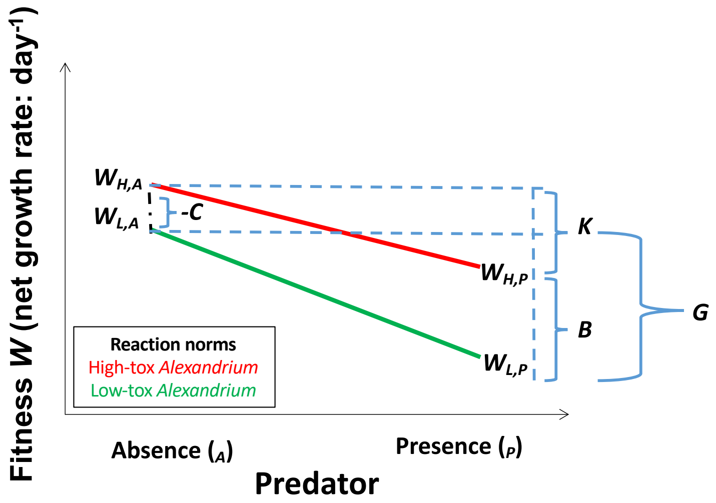

Graphical representation of the modification of Simms and Rausher's (1987) and Simms (1992) models, illustrated for the lowly and highly toxigenic strains. The model was modified to account for observations (-*C*, +*K*). Refer to the model terms in Fig. 1.

S4.

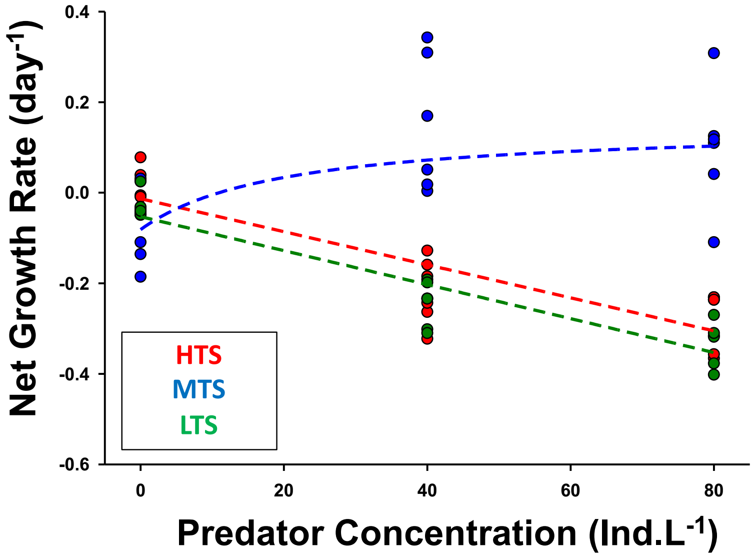

Net growth rate versus predator concentration of three strains of *Alexandrium catenella* under low-light intensity (~10 µmol m^{-2} s^{-1}) condition (n=5 per predator treatment). Except for the light intensity and volume (50 ml), other experimental methods are like the experiments at the high-light intensity shown in Fig. 5A.

S5.

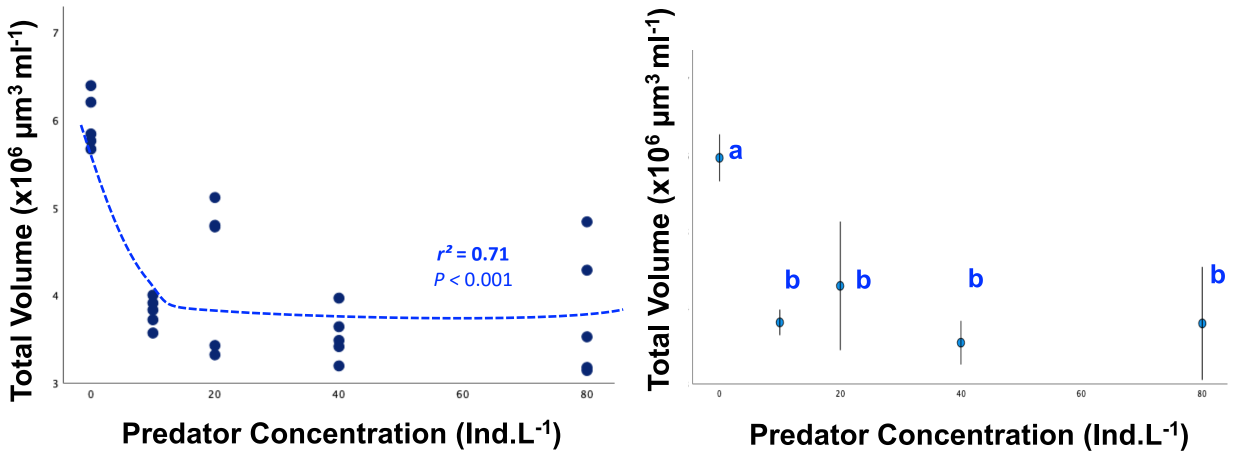

Total volume of the MTS using the mean cell number, (Ct-Co)/2, where Ct and Co are the final and initial cell concentrations. Line is regression fit and letters next to bars represent significant statistical differences between mean values of groups compared to control (0 ind. L^{-1}) and among treatments. Error bars represent ± 1 standard deviation of the mean (n=5).
